# Promotion of Cell Death in Cisplatin-Resistant Ovarian Cancer Cells through KDM1B-DCLRE1B Modulation

**DOI:** 10.3390/ijms20102443

**Published:** 2019-05-17

**Authors:** Yeon Kyu Lee, Jinyeong Lim, So Young Yoon, Jong Cheon Joo, Soo Jung Park, Yoon Jung Park

**Affiliations:** 1 Department of Nutritional Science and Food Management, Ewha Womans University, Seoul 03760, Korea; yeonkyulee92@gmail.com (Y.K.L.); aster1217@gmail.com (J.L.); syyoon0504@gmail.com (S.Y.Y.); 2 Department of Sasang Constitutional Medicine, College of Korean Medicine, Wonkwang University, Iksan 54538, Korea; jcjoo@wku.ac.kr; 3 Department of Sasang Constitutional Medicine, College of Korean Medicine, Woosuk University, Jeonju 55338, Korea

**Keywords:** ovarian cancer, *Oldenlandia diffusa*, cisplatin, cell death, KDM1B, DCLRE1B

## Abstract

Ovarian cancer is the gynecological malignancy with the poorest prognosis, in part due to its high incidence of recurrence. Platinum agents are widely used as a first-line treatment against ovarian cancer. Recurrent tumors, however, frequently demonstrate acquired chemo-resistance to platinum agent toxicity. To improve chemo-sensitivity, combination chemotherapy regimens have been investigated. This study examined anti-tumor effects and molecular mechanisms of cytotoxicity of *Oldenlandia diffusa* (OD) extracts on ovarian cancer cells, in particular, cells resistant to cisplatin. Six ovarian cancer cells including A2780 and cisplatin-resistant A2780 (A2780cis) as representative cell models were used. OD was extracted with water (WOD) or 50% methanol (MOD). MOD significantly induced cell death in both cisplatin-sensitive cells and cisplatin-resistant cells. The combination treatment of MOD with cisplatin reduced viability in A2780cis cells more effectively than treatment with cisplatin alone. MOD in A2780cis cells resulted in downregulation of the epigenetic modulator KDM1B and the DNA repair gene DCLRE1B. Transcriptional suppression of KDM1B and DCLRE1B induced cisplatin sensitivity. Knockdown of KDM1B led to downregulation of DCLRE1B expression, suggesting that DCLRE1B was a KDM1B downstream target. Taken together, OD extract effectively promoted cell death in cisplatin-resistant ovarian cancer cells under cisplatin treatment through modulating KDM1B and DCLRE1B.

## 1. Introduction

*Oldenlandia diffusa* (Willd.) Roxb. (OD) is a member of the Rubiaceae Family, and is well known as a medicinal plant in China [1,2]. The plant is used for treating hepatitis, tonsillitis, rheumatism, arthritis, autoimmune disease, and tumors of the liver, lung, and stomach [3]. It contains bioactive compounds, such as pentacyclic triterpenoid acids, including ursolic and oleanolic acids. Ursolic acid and oleanolic acid have been reported to have anti-tumor, apoptotic, antioxidant, cytotoxic, and anti-angiogenic activity, and anti-inflammatory effects [4,5]. Extracts of OD have also been reported to have anticancer effects [6,7]. Treatment of human breast cancer MCF-7 cells with OD extracts induced cell death through increased expression and activation of apoptosis-related proteins [8]. For colorectal cancer, aqueous OD extracts inhibited tumor growth both in vitro and in vivo via activation of p53 [9]. OD anti-tumor outcomes have been reported in several cancer studies, but its effects and apoptotic mechanisms have not been reported for ovarian cancer.

Ovarian cancer is one of the most common types of gynecological malignant tumors. In 2012, 238,700 cases and 15,900 deaths were reported worldwide [10]. Due to difficulties in early detection of ovarian cancer symptoms, most patients are diagnosed with late stage disease. Subsequent recurrence rates are high (70%), and acquired resistance to drug treatment results in high mortality [11].

Cisplatin is a first-line platinum-based drug used for the treatment of ovarian cancer. It causes DNA damage that induces cell apoptosis in malignant cells [12]. Among different types of DNA damage, DNA inter-strand crosslinks (ICL) are notable for inducing tumor cell death [13]. ICLs impede DNA replication and cause replication fork collapse and DNA double-strand breaks [14]. In mammalian cells sensitive to nitrogen mustard 1B/, DNA cross-link repair 1B (SNM1B/DCLRE1B) plays an important role in the repair system for ICL-mediated DNA damage [14]. Deficiency or inhibition of DCLRE1B in mouse fibroblast and human lymphoma cells reduces cell viability after cisplatin treatment [13].

Epigenetic changes that modulate gene expression without altering DNA sequences are reported as signatures of tumorigenesis and aggressive progression in various malignancies, including ovarian cancer. Aberrant methylation patterns in DNA and lysine residues of histones have been reported in ovarian cancer [15]. Lysine-specific demethylase 1 (LSD1/KDM1A) is a histone demethylase that removes mono- and dimethyl-lysine 4 of histone H3 (H3K4me1/2) [16] and is overexpressed in various cancer types including breast, lung, and prostate cancer [17]. Inhibition of its activity induces apoptosis and autophagy in SKOV3 ovarian cancer cells [18]. In addition, LSD2/KDM1B, which share similar domain homology with KDM1A, demethylates H3K4me1/2 and H3K9m21/2 and its knockdown causes death of breast cancer cells [19]. Also, treatment with bioactive compounds of OD induces changes in epigenetic mechanisms. Oral administration of ursolic acid reduces inflammation by inhibiting epigenetic modifiers including DNA methyltransferases (DNMTs) and histone deacetylases (HDACs) in leukocytes [20]. Based on this understanding, we investigated anti-tumor effects, and a potential molecular mechanism of OD extracts on ovarian cancer cells.

## 2. Results

### 2.1. Combination Treatment with Cisplatin and O. diffusa Extracts Reduces Cell Viability

**Figure 1:**
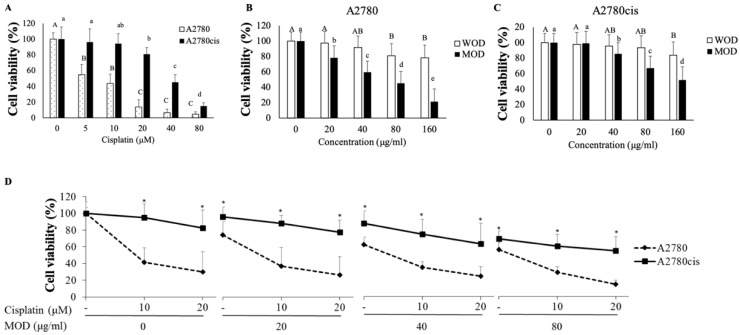
Cisplatin and OD affect cell viability of ovarian cancer cells. Cell viability was measured after treatment with (**A**) cisplatin (0–80μM), water (WOD) and methanol (MOD) extract of OD in (**B**) A2780 and (**C**) A2780cis, (**D**) combination of cisplatin (0–20 μM) and MOD (0–80 μg/mL) for 48 h on both ovarian cancer cells A2780 and A2780cis. To measure cell viability, 3-(4,5-dimethylthiazol-2-yl)-2,5-diphenyltetrazolium bromide (MTT) assays were used. Data are shown as the mean ± standard deviations (STDEV). Statistically, one-way analysis of variance (ANOVA) and Duncan’s post-hoc tests were used for the comparison between treatment doses in each cell line. Different letters indicate significant difference within a group as determined by Student’s t-test for A2780 (capital letters) and A2780cis (small letters) results. * *p* < 0.05 was used to define statistical significance.

Firstly, cell viability was determined as resistance indicators for A2780 cell lines, A2780 and A2780cis. Viability of A2780 cells was significantly reduced by nearly 50% by 5 μM cisplatin treatment (Figure 1A); however, cell viability of A2780cis was not significantly reduced until cisplatin was 20 μM, which indicated resistance to cisplatin.

Both *O. diffusa* extracts, WOD and MOD, affected cell viability in A2780 (Figure 1B). Compared to WOD, MOD treatment reduced cell viability depending on concentration. Only the highest concentration of WOD, 160 μg/mL, affected cell viability of A2780cis; MOD treatment had a significantly greater impact on cell viability (Figure 1C). A2780cis cell viability was reduced to 80% at a MOD concentration of 40 μg/mL and decreased to nearly 60% at 160 μg/mL. In comparison with cisplatin or WOD treatment, MOD treatment showed a significantly greater impact on cell viability of A2780cis cells

Combined treatment with cisplatin and MOD induced a synergistic effect on cell viability of A2780cis (Figure 1D). Cisplatin showed a dramatic difference in toxicity to A2780 and A2780cis, more than 50–60% cell viability at 10–20 μM. MOD alone also led to a reduction in cell viability, but with relatively less different between A2780 and A2780cis. Combined treatment with MOD and cisplatin effectively reduced cell viability even in A2780cis. MOD at concentrations of 20, 40, and 80 μg/mL with cisplatin (10 μM) decreased differences in cell viability in A2780 and A2780cis cells to 60, 40, and 10%, respectively, indicating that using MOD in combination with a low concentration of cisplatin might be a possible strategy for overcoming resistance to cisplatin.

### 2.2. MOD Treatment Induces Apoptosis in Ovarian Cancer Cells

**Figure 2:**
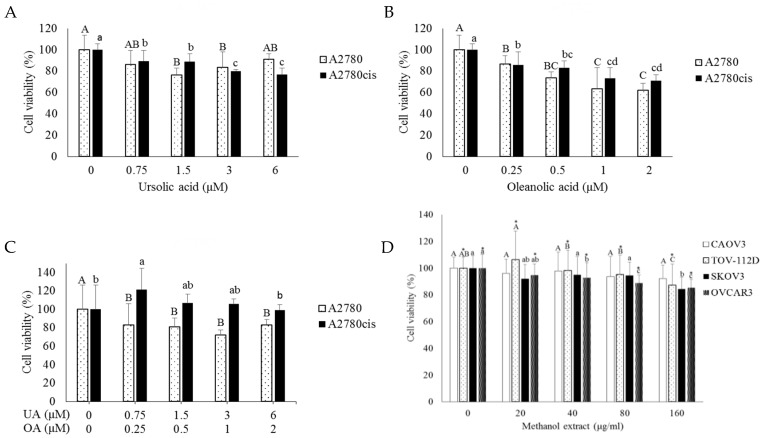

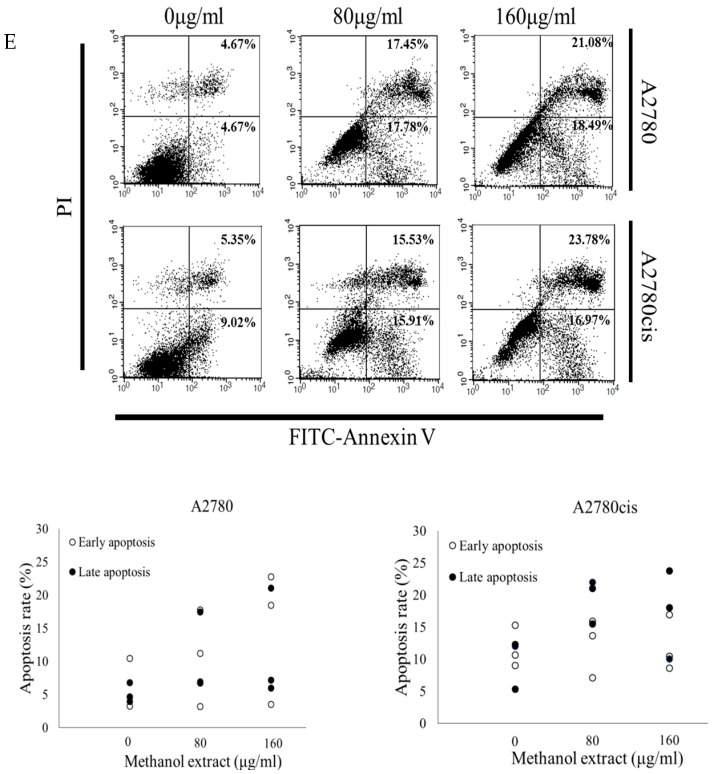
MOD affects ovarian cancer cell viability to a greater extent than individual bioactive compounds. Effects of bioactive compounds of OD on A2780 and A2780cis cells: (**A**) Ursolic acid (UA), (**B**) oleanolic acid (OA), and (**C**) UA and OA in combination. (**D**) Cisplatin-sensitive cell lines (CAOV3, TOV-112D) and -resistant cell lines (SKOV3, OVCAR3) were treated with methanol extract of OD (MOD). Cell viability was measured with an MTT assay after 48-h treatment. Each value uses the non-treated group as 100%. Oneway ANOVA analysis, followed by Duncan post-hoc test, was used for the comparison between treatment doses in each cell line. Different letters indicate significant difference inside a group (*p* < 0.05). (**E**) Apoptosis was inducted after a 48-h MOD treatment at various concentrations (0, 80, and 160 μg/mL) in both A2780 and A2780cis cells, stained using fluorescein isothiocyanate (FITC)-conjugated annexin-V and propidium iodide (PI), and subjected to flow cytometric analysis. Representative results are shown. In the bottom panels, the left panel is the result from A2780 cells, the right panel is that from A2780cis cells. Different letters indicate significant difference inside a group as determined by Student’s t-test for A2780 (capital letters) and A2780cis (small letters) results.

Previously known for its bioactivities of OD, ursolic acid (UA) and oleanolic acid (OA) were quantified in WOD and MOD using high-performance liquid chromatography (HPLC) (Table 1). UA and OA were more abundant in MOD, 15.905 mg/g and 5.365 mg/g, respectively, compared to WOD, 0.187 mg/g, and 0.054 mg/g, respectively. Since WOD had relatively less effect on cell viability for both A2780 and A2780cis cells, we tested the effects of UA and OA on cell viability using concentrations equivalent to levels measured in MOD. UA, 0, 0.75, 1.5, and 3.6 μM, and OA, 0, 0.25, 0.5, 1, and 2 μM correspond to concentrations in MOD of 0, 20, 40, 80, and 160 μg/mL. UA treatment did not influence cell viability even at the highest concentration (Figure 2A). OA and combination treatment reduced cell viability of A2780 cells to 60% and 90%, respectively, at the highest concentration (Figure 2B); this effect was not as great as effects induced by MOD treatment (Figure 2C). In A2780cis cells, UA and OA alone reduced cell viability at the highest concentrations, but to the lesser extent compared to MOD, while the combination did not affect cell viability (Figure 2C). The results indicate that the effect of MOD is not dependent only on UA and OA. For further analysis, we used MOD itself rather than individual substances.

To confirm the effectiveness of MOD, an additional four ovarian cancer cells were evaluated; CAOV3, TOV-112D as cisplatin-sensitive ovarian cancer cells (CSC) and SKOV3, OVCAR3 as cisplatin-resistant ovarian cancer cells (CRC) (Figure 2D). TOV-112D viability was reduced in a dose-dependent manner from 40 μg/mL, while CAOV3 did not respond to MOD treatment. Similarly, OVCAR3 cell viability decreased in a dose-dependent manner, while SKOV3 viability was reduced only at the highest concentration. Overall, cell viability declined with MOD treatment regardless of cisplatin resistance, although each cell line had slightly different sensitivity to MOD. It is noteworthy that the effect in these cell lines were not as effective as that in A2780 lines, indicating that different doses and periods of MOD treatment might be required for each cell line.

MOD treatment-induced cell death occurred through apoptosis (Figure 2E). In the quadrant graph, the lower left indicates viable cells, the lower right, early apoptotic cells and the upper right, late apoptotic cells. As MOD concentration increased, numbers of early and late apoptotic cells increased, in both A2780 and A2780cis cells. A2780 apoptotic cells increased from 9.34% to 39.57% at concentrations of 0 μg/mL and 160 μg/mL, respectively. Apoptosis was also induced in A2780cis, increasing from 14.37% to 40.75% at concentrations of 0 μg/mL and 160 μg/mL, respectively. However, apoptosis levels confirmed by three independent experiments were variable, although early and late apoptotic cells tended to increase compared with untreated control cells.

### 2.3. Downregulation of KDM1B Induces Cisplatin Sensitivity in CRC

**Figure 3:**
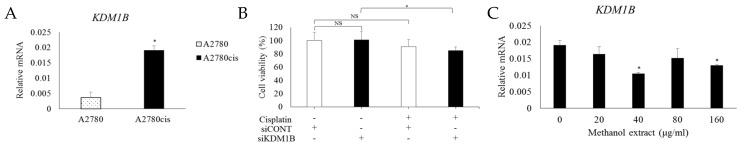
Cisplatin sensitivity increases in CRC after *KDM1B* downregulation. (**A**) *KDM1B* mRNA expression quantified in A2780 and A2780cis cells. (**B**) Transfection of siCONT and siKDM1B in A2780cis cells, followed by treatment with cisplatin for 48 h. Cell viability was measured with MTT assays. Following treatment with MOD (0 and 160 μg/mL) for 48 h, (**C**) *KDM1B* mRNA expression as the mean ± STDEV. Student’s t-test was used to assess differences between non-treated and treated group and between A2780 and A2780cis cells. * *p* < 0.05 was used to define statistical significance. NS: not significant.

To identify differences between CSC and CRC, we focused on epigenetic modulators. According to a previous study analyzing epigenetic modulators relevant to cisplatin resistance [21], *KDM1B* was a differentially expressed gene (DEG) with the highest fold change in CRC. To validate the microarray result, KDM1B mRNA expression was measured through quantitative reverse transcriptase PCR (RT-PCR) (Figure 3A). A2780cis cells showed significantly higher expression of *KDM1B* than A2780 cells. We further investigated whether KDM1B was involved in cisplatin sensitivity of ovarian cancer cells. Data show that the *KDM1B* gene could be key to gaining cisplatin resistance. KDM1B has been manipulated and cell viability was measured in cisplatin-resistant cells, A2780cis.

To confirm the KDM1B effect at the RNA level, small interfering RNA (siRNA) was used for knock-down expression of *KDM1B* in A2780cis cells. Treatment with cisplatin alone at 10 uM or siKDM1B knock-down did not affect cell viability (Figure 3B). However, siRNA knock-down of *KDM1B* followed by cisplatin treatment showed a significant reduction in cell viability. These results indicate that inhibition of *KDM1B* expression helps overcome cisplatin resistance cisplatin-resistant ovarian cancer cells resulting in increased sensitivity to drug toxicity and decreased tumorigenesis.

To investigate whether KDM1B is involved in MOD-induced cell death in CRC, *KDM1B* gene expression was measured in A2780cis cells after MOD treatment. *KDM1B* mRNA levels significantly decreased at MOD concentrations of 40 μg/mL and 160 μg/mL (Figure 3C), suggesting *KDM1B* expression might be involved in the mechanism for MOD effects.

### 2.4. Downregulation of DCLRE1B is a Factor of CRC Death

**Figure 4:**
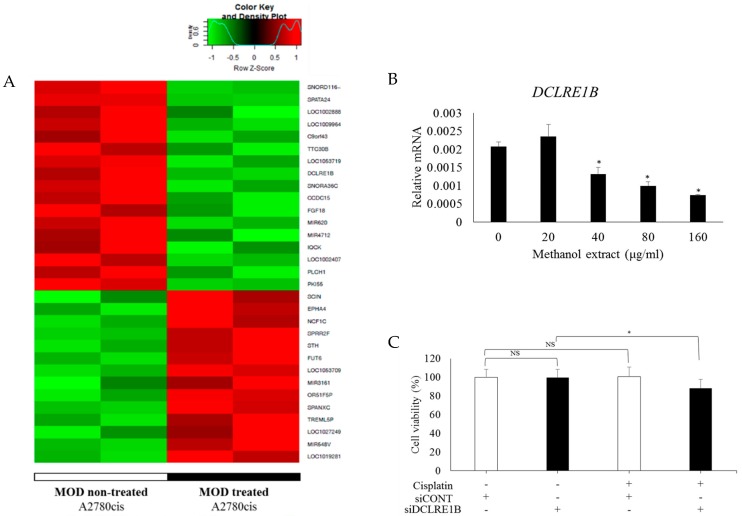
MOD treatment increases the sensitivity of cisplatin in CRC through downregulation of *DCLRE1B*. (**A**) Differentially expressed genes (DEGs) between non-treated and MOD treated A2780cis cells were represented in a heatmap. The 31 genes were identified as DEGs. Expression of 14 genes increased and 17 genes decreased in MOD treated cells using a cutoff standard of FC > |1.5| and *p*-value < 0.05. After treatment of A2780cis cells with MOD in various concentration (0 and 160 μg/mL) for 48 h, (**B**) *DCLRE1B* mRNA expression, (**C**) transfection of A2780cis with control siRNA (siCONT) and DCLRE1B-targeting siRNA (siDCLRE1B) followed by cisplatin treatment for 48 h. Cell viability was measured with MTT assays. Data are shown as the mean ± STDEV. Significant differences between non-treated and treated groups was assessed using the Student’s t-test. * *p* < 0.05 was used to define statistical significance. NS: not significant.

To identify of KDM1B as a target for the effects of MOD treatment in an unbiased way, genome-wide expression analysis was conducted using a microarray four MOD non-treated and treated A2780cis cells. Using a cutoff standard as fold change (FC) > |1.5| and a raw *p*-value < 0.05, 31 DEGs were detected, including 14 upregulated and 17 downregulated genes in MOD treated cells (Figure 4A). The *DCLRE1B* gene was significantly downregulated. This downregulation was confirmed through quantitative RT-PCR (Figure 4B).

siRNA-mediated repression of *DCLRE1B* altered cisplatin resistance in A2780cis cells (Figure 4C). Treatment with cisplatin at 10 uM or siRNA knock-down of *DCLRE1B* did not affect the viability of A2780cis cells. However, siRNA knock-down of *DCLRE1B* followed by treatment with cisplatin led to a significant reduction in viability of these cells. In short, by downregulating *DCLRE1B* and *KDM1B* gene expression, drug sensitivity increases in CRC, which become susceptible to cisplatin-induced cell death.

### 2.5. MOD Treatment Induced Cell Death of CRC by Regulating KDM1B and DCLRE1B Expression

**Figure 5:**
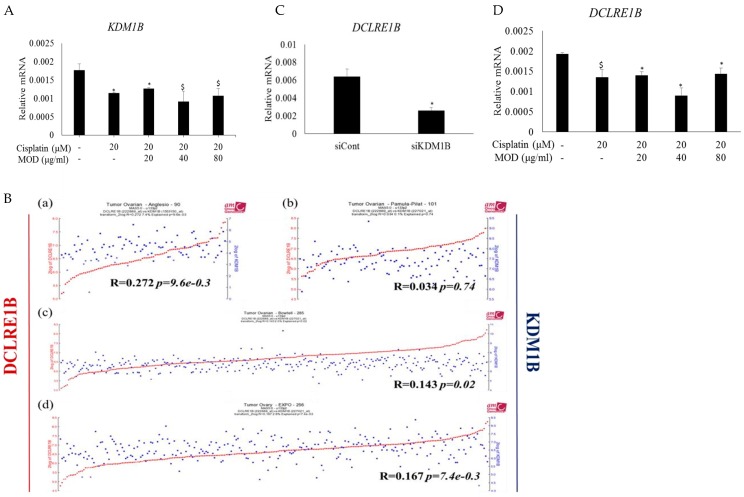
MOD enhancement of cisplatin sensitivity is correlated with KDM1B and DCLRE1B expression. Treatment of A2780cis cells with a combination of cisplatin and various concentrations of MOD for 48 h: (**A**) *KDM1B* and (**D**) *DCLRE1B* mRNA expression. (**B**) Correlation graphs between *DCLRE1B* and *KDM1B* gene expression from R2: microarray analysis and visualization platform (Available at: http://r2.amc.nl) using cohorts from four different ovarian cancer patient datasets: (**a**) Anglesio-90, (**b**) Pamula-Pilat-101, (**c**) Bowtell-285 and (**d**) EXPO-256. (**C**) A2780cis were transfected with siCONT and siKDM1B for 24 h and *DCLRE1B* mRNA expression was subsequently quantified. Data are shown as mean ± STDEV. Each value is based on using non-treated or siCONT groups as 100%. Significant differences between siCONT and siKDM1B or non-treated and treated groups were assessed using Student’s t-test. * *p* < 0.05 and ^$^ *p* < 0.07 were used to define statistical significance.

Combined treatment with cisplatin and MOD efficiently induced cell death in cisplatin-resistant ovarian cancer cells (Figure 1D). Thus, we tested whether combined treatment altered *KDM1B* mRNA expression. Treatment with cisplatin and MOD together, decreased *KDM1B* expression at concentrations of 20, 40 and 80 μg/mL (Figure 5A). Compared to treatment with MOD alone (Figure 3C), combined treatment downregulated *KDM1B* mRNA expression more efficiently.

To investigate a functional link between KDM1B and DCLRE1B, and MOD treatment, we examined gene expression patterns using the R2 platform (Available at: http://r2.amc.nl) based on four cohort datasets of ovarian cancer patients. *KDM1B* expression was highly correlated with *DCLRE1B* expression (Figure 5B). Three of the four human ovarian cancer patient data sets, including (a) Tumor Ovarian-Anglesio, (c) Tumor Ovarian-Bowtell and (d) Tumor Ovarian-Expo data sets, showed this KDM1B and DCLRE1B correlation with *R* square < 1 and *p*-value < 0.05.

More direct evidence came from analysis of *DCLRE1B* expression upon *KDM1B* repression (Figure 5C). siRNA-mediated inhibition of *KDM1B* expression in A2780cis cells led to downregulation of *DCLRE1B* expression, which was consistent with the correlation analysis on cohorts (Figure 5B). *DCLRE1B* expression is correlated with *KDM1B* expression, and *DLCRE1B* mRNA expression was measured in A2780cis cells treated with MOD and cisplatin. DLCRE1B mRNA expression was significantly reduced (Figure 5D), as was *KDM1B* mRNA expression (Figure 5A).

## 3. Discussion

In the present study, we show that OD extracts induce cell death via apoptosis not only in CSC, but also in CRC. MOD was more efficient in inducing cell death compared to WOD. In particular, the combination of cisplatin and MOD more effectively killed cells, thus reducing resistance to cisplatin toxicity in CRC. The effects were, at least in part, mediated through KDM1B-induced downregulation of *DCLRE1B*.

MOD treatment was more effective at reducing the viability of ovarian cancer cells than WOD treatment. This result is consistent with a previous breast cancer study that showed no effect of WOD compared to OD extracted with methanol or butanol [8]. Based on HPLC analysis, MOD has 15 times and 5 times greater amounts of UA and OA, respectively, compared to WOD. OD extract has been reported to include asperuloside, E-6-O-p-coumaroyl scandoside methyl ester, and E-6-O-p-coumaroyl scandoside methyl ester-10-methyl ether, in addition to UA and OA [6]. Individual and combined treatment with UA and OA showed relatively mild cytotoxic effects compared to MOD itself, suggesting that other compounds in MOD might contribute to MOD effects on cell viability.

CRC appeared more sensitive to MOD when treated along with cisplatin. Combined treatment with cisplatin and MOD narrowed the gap in cell viability results between A2780 and A2780cis. As MOD concentrations increased, cytotoxicity of cisplatin to A2780 and A2789cis cells became more similar. Such synergistic effects of combining compounds with cisplatin have also been reported in treated human non-small cell lung cancer NCI-H23 cells as well as ovarian cancer A2780cis cells [22,23].

OD compounds have been reported to act via epigenetic mechanisms resulting in anti-inflammatory, anti-tumor and apoptotic effects [20], and we focused on epigenetic changes caused by OD treatment. In a previous study, 27 DEGs that encode epigenetic modulators were identified in comparisons of four CRCs and four CSCs [21]. KDM1B, which had the highest fold change in array analysis, showed significantly higher expression in A2780cis than A2780 cells. siRNA-mediated knockdown of *KDM1B* did not affect the viability of A2780cis cells, but its combined treatment with cisplatin significantly reduced cell viability. Also, *KDM1B* mRNA expression was downregulated in MOD-treated A2780cis cells. This result indicates that MOD might induce cisplatin sensitivity by downregulation of *KDM1B*.

In our study, DCLRE1B was identified as a DEG between MOD-treated and non-treated A2780cis cells through genome-wide expression analysis using a microarray. DCLRE1B is required for repair of ICL-related DNA damage and its deficiency increases sensitivity to cisplatin treatment [13,24]. Pathologically, *DCLRE1B* gene alterations are unfavorable for renal, liver, and pancreatic cancer (Figure S1). Moreover, it has been demonstrated that CRC removed cisplatin-caused ICL 2.5 times more rapidly than CSC, even though the level of cisplatin-mediated ICL was much higher in CRC [25]. Removal of ICL has been suggested to be due to increased ICL repair efficiency in CRC [26]. Consistent with cell viability findings, MOD treatment of A2780cis cells led to downregulation of *DCLRE1B* mRNA levels. Also, siRNA-mediated knockdown of *DCLRE1B* increased sensitivity to cisplatin treatment, resulting in the death of A2780cis cells.

The function of KDM1B is demethylation of H3K9me2 and H3K9me3, both known as transcriptionally repressive markers [18]. In particular, H3K9me3, which compacts chromatin structure, is considered to inhibit DNA damage repair [27]. Although direct evidence is insufficient to confirm the relationship between KDM1B activity and DNA damage repair, KDM1B might help cisplatin-resistant cancer cells to reduce chromatin compacting, and thus maintain transcriptional activation of DNA repair genes including *DCLRE1B*. A correlation between *KDM1B* and *DCLRE1B* expression was found in human ovarian cancer cohort studies. Additional strong correlations between the two genes is shown in the present study. siRNA-mediated *KDM1B* knock-down resulted in downregulation of *DCLRE1B* mRNA expression in A2780cis cells.

In conclusion, our results demonstrate the potential of MOD for synergic treatment with cisplatin to overcome resistance to cisplatin in CRC by modulating epigenetic regulation by KDM1B on the DNA damage repair gene DCLRE1B. Further investigation is needed to investigate a direct relationship between OD and KDM1B-mediated downregulation of *DCLRE1B*. Additionally, it also requires evidence from in vivo studies with a long-term treatment for clinical perspectives.

## 4. Materials and Methods

### 4.1. Cell Culture

Six ovarian cancer cell lines (A2780, CAOV3, TOV-112D, A2780cis, OVCAR-3, SKOV-3) were classified for their cisplatin resistance, using a previous report [28]. A2780, CAOV3 and TOV-112D were categorized as cisplatin-sensitive (CSC) and A2780cis, OVCAR-3, and SKOV-3 as cisplatin-resistant (CRC). A2780, A2780cis, and OVCAR-3 cells were cultured in RPMI1640 medium (Welgene, LM 011-03, Daegu, South Korea). CAOV3 and SKOV-3 cells were cultured in Dulbecco’s Modified Eagle’s Medium (Welgene, LM 001-05, Daegu, South Korea) and McCoy’s 5a (Gibco, 16600-082, Gaithersburg, MD, USA) respectively. TOV-112D cells were cultured in MCDB105 (Welgene, LM 016-50, Daegu, South Korea) and Medium199 (Gibco, 11150-059, Gaithersburg, MD, USA) in 1:1 proportion. All culture media were supplemented with 10% FBS (Atlas, Fort Collins, CO, USA), 100 UmL penicillin and 100 μ/mL streptomycin, and maintained at 37 °C and 5% CO_2_. Additionally, for culturing A2780cis, cisplatin (Sigma, St. Louis, MO, USA), 100 μM, was added to the medium every 2–3 passages to maintain cisplatin resistance.

### 4.2. Preparation of O. diffusa Extract

An extract of whole dried herb *O. diffusa* was obtained from Hanpoong Pham & Foods Co., Ltd. (Jeonju, Korea). OD was extracted with water (WOD) or 50% of methanol (MOD). Extracts were dissolved in phosphate-buffered saline (PBS) as a stock solution (250 mg/mL final concentration) and stored in −20 °C for further use.

### 4.3. High-Performance Liquid Chromatography (HPLC)

The *O. diffusa* (OD) extract was analyzed with high-performance liquid chromatography (HPLC) (Hanpoong Pham & Foods Co., Ltd., Jeonju, Korea). OD was extracted with water (WOD) or 50% of methanol (MOD). Nine-hundred g of OD was carefully selected and extracted twice with 20 volumes of water or 50% of methanol for 3 h at 100 °C or 86 °C, respectively. The extracts were filtered through a 1-micron filter and vacuum evaporated dryness. The amount of dried water and 50% methanol extracts were 106.2 g with 11.8% yield and 95.3 g with 10.59% yield, respectively. HPLC used HPLC grade acetonitrile (ACN): Methanol 80:20, a capillary voltage of 3 kV, a wavelength of 210 nm, column size of 25 cm and flow rate of 0.15 mL/min for 30 min.

### 4.4. MTT Assay for Cell Viability

Cells were seeded in 96-well plates. A2780 and A2780cis cells were seeded with 1 × 10^4^ cells per well and other cell lines were seeded with 9 × 10^3^ cells per well. Cells were incubated in 37 °C for 24 h. WOD, MOD, UA, OA, and cisplatin were added in various concentrations, and incubation continued for 48 h. After treatment, 15 μL 3-[4,5-dimethylthiazol-2-yl]-2,5-diphenyltetrazoliumbromide (MTT) (Sigma Aldrich, St, Louis MO, USA) stock solution in PBS (5 mg/mL) was added to each well. After 4 h at 37 °C, the medium was removed, and cells were solubilized in 100 μL dimethyl sulphoxide (DMSO). The viability of cells was read using a microplate reader (Biochrome, Berlin, Germany) at 562 nm.

### 4.5. Apoptotic Detection by Flow Cytometry

A2780 and A2780cis cells were seeded in six-well plates with 5 × 10^5^ cells per well and treated with MOD in various concentration (0, 80, and160 μg/mL), and incubated at 37 °C for 48 h. Treated cells were harvested, trypsinized and washed with PBS. To detect apoptotic cells, cells were incubated in the dark at room temperature for 15 min with either Annexin V-FITC or propidium iodide (BD Bioscience, CA, USA) for assessment of early and late apoptotic stages, respectively. Flow cytometry (FACS Calibur flow cytometer, Becton Dickinson and Company, BD Biosciences, CA, USA) using data acquisition software (BD CellQuest Pro software, version 6.0).

### 4.6. Microarray and Data Analysis

For microarray analysis, four samples were prepared in duplicate. A2780 and A2780cis were treated with or without 80 μg/mL MOD for 48 h and RNA was isolated. Microarray analysis was performed using Macrogen Co., Ltd. (Seoul, Korea) and the Affymetrix whole transcript expression array. Briefly, cDNA was synthesized using the GeneChip WT amplification kit (Affymetrix, Santa Clara, CA, USA). The sense cDNA was then fragmented and biotin-labeled with TdT (terminal deoxynucleotidyl transferase) using the GeneChip WT terminal labeling kit (Affymetrix, Santa Clara, CA, USA). Approximately 5.5 μg of labeled target DNA was hybridized to the Affymetrix GeneChip Human 2.0 ST Array (Affymetrix, Santa Clara, CA, USA) at 45 °C for 16 h. Hybridized arrays were washed and stained on a GeneChip Fluidics Station 450 (Affymetrix, Bedford, MA, USA) and scanned on a GCS3000 Scanner (Affymetrix, Bedford, MA, USA). Signal values were computed using the Affymetrix^®^ GeneChip™ Command Console software (Affymetrix^®^ GeneChip™ Command Console software). All data analysis and visualization of differentially expressed genes was conducted using the Affymetrix^®^ GeneChip™ Command Console software R 3.1.2. A cutoff standard was established as |FC| > 1.5 and a raw *p*-value < 0.05 was used for assessing statistical significance.

### 4.7. siRNA Transfection Assay

A2780 and A2780cis cells, 3 × 10^4^ cells per well, were seeded in 96-well plates in regular growth medium and incubated at 37 °C for 24 h. Transfection reagent DharmaFECT1 (Dharmacon, Thermo Scientific, Lafayette, CO, USA) and siGENOME human KDM1B siRNA or siGENOME human DCLRE1B siRNA were transfected into cells. After 24 h, transfection efficiency and cell viability were confirmed. The medium was then replaced with fresh medium that contained 10 μM cisplatin and incubation continued for 48 h. Cell viability was then assessed using an MTT assay.

### 4.8. Reverse Transcription and Quantitative RT-PCR

Cells were seeded in 6-well plates with 5 × 10^5^ cells per well and treated with MOD or cisplatin for 48 h. Total RNA was extracted using TRIzol reagent (Ambion, Carlsbad, CA, USA) and isopropanol precipitation. Complementary DNA (cDNA) synthesis, converting one μg of total RNA, was performed using oligo dT primer and RevertAid Reverse Transcriptase (Thermo Fisher Scientific, Waltham, MA, USA). Template and solution including SYBR Green PCR Master Mix (Qiagen, Valencia, CA, USA) and primers were used for quantitative RT-PCR (qPCR) which was performed with a Rotor Gene Q machine (Qiagen, Hilden, Germany). Primers used were: *Glyceraldehyde 3-phosphate dehydrogenase* (*GAPDH*) forward, 5′- GAAGGTGAAGGTCGGAGTCA-3′ and reverse, 5′-CATGGGTGGAATCATATTGGA-3′; *KDM1B* forward, 5′-GCGTGCTGATGTCTGTGATT-3′ and reverse, 5′-TTGTGGGATCTGGGACCTC-3′; *DCLRE1B* forward, 5′-CAACACCAATTGCAATCCAG-3′ and reverse, 5′-AGCCTTCTCCTCCACTGTGA-3′. qPCR protocol was 95 °C for 5 min, with 40 cycles at 95 °C for 5 s and 60 °C for 10 s. Each sample was analyzed in duplicate and expression values were normalized against GAPDH.

## Figures and Tables

**Table 1:** Quantification of standard substances, ursolic acid, and oleanolic acid, in *O. diffusa* extracts using HPLC analysis.

Extract	Concentration of Treatment	Ursolic Acid	Oleanolic Acid	Units
	Water extract	0.187	0.054	mg/g
	0	0	0	
	20	0.00374	0.00108	
Water extract	40	0.00748	0.00216	µg/mL
	80	0.01496	0.00432	
	160	0.02992	0.00864	
	Methanol extract	15.905	5.365	mg/g
	0	0	0	
	20	0.3181	0.1073	
Methanol extract	40	0.6462	0.2146	µg/mL
	80	1.2724	0.4292	
	160	2.5448	0.8584	

## Supplementary Materials

Supplementary materials can be found at https://www.mdpi.com/1422-0067/20/10/2443/s1, Figure S1: Pathology aspect of *DCLRE1B* gene.

## Abbreviations

ACN	Acetonitrile
cDNA	Complementary DNA
CRC	Cisplatin-resistant ovarian cancer cells
CSC	Cisplatin-sensitive ovarian cancer cells
DEG	Differentially expressed gene
DMSO	Dimethyl sulphoxide
DNMTs	DNA methyltransferases
GAPDH	Glyceraldehyde 3-phosphate dehydrogenase
HDACs	Histone decaetylases
HPLC	High-performance liquid chromatography
H3K4me1/2	Mono- and dimethyl-lysine 4 of histone H3
ICL	Inter-strand crosslinks
LSD1/KDM1A	Lysine-specific demethylase 1
MOD	Methanol extract of OD
MTT	3-[4,5-dimethylthiazol-2-yl]-2,5-diphenyltetrazoliumbromide
PBS	Phosphate-buffered saline
PI	Propidium iodide
OA	Oleanolic acid
OD	*Oldenlandia diffusa*
SNM1B/DCLRE1B	DNA cross-link repair 1B
UA	Ursolic acid
WOD	Water extract of OD
