# Supplementary material for: Promotion of Cell Death in Cisplatin-Resistant Ovarian Cancer Cells through KDM1B-DCLRE1B Modulation
Source: Int J Mol Sci. 2019 May 17;20(10):2443. doi: 10.3390/ijms20102443 (PMC6566920; doi:10.3390/ijms20102443)
Supplement: Supplementary file 1 [file ijms-20-02443-s001.pdf]

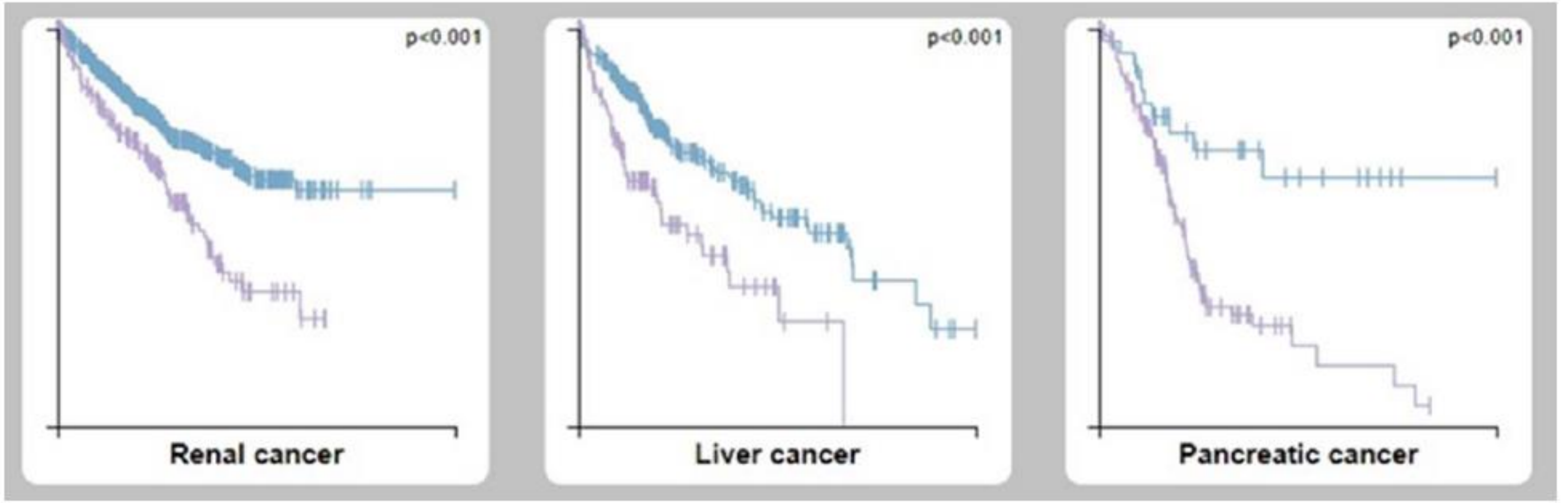

Figure S1. Pathology aspect of DCLRE1B gene. Significant association ($p < 0.01$) with patient survival rate between cancers and high expression of DCLRE1B are shown as Kaplan-Meier plots using pathology data from protein atlas (v17.proteinatlas.org). In renal, liver and pancreatic cancer, prognosis shows as unfavourable. Whether the prognosis is favourable or unfavourable is indicated in brackets.
